# Clinically translatable gold nanozymes with broad spectrum antioxidant and anti-inflammatory activity for alleviating acute kidney injury

**DOI:** 10.7150/thno.66518

**Published:** 2021-10-17

**Authors:** Dong-Yang Zhang, Tianhui Tu, Muhammad Rizwan Younis, Kathy S. Zhu, Hengke Liu, Shan Lei, Junle Qu, Jing Lin, Peng Huang

**Affiliations:** 1 Marshall Laboratory of Biomedical Engineering, International Cancer Center, Laboratory of Evolutionary Theranostics (LET), School of Biomedical Engineering, Shenzhen University Health Science Center, Shenzhen 518060, China.; 2 Key Laboratory of Optoelectronic Devices and Systems of Ministry of Education and Guangdong Province, College of Optoelectronic Engineering, Shenzhen University, Shenzhen 518060, China.; 3 National Clinical Research Center for Oral Diseases, National Engineering Laboratory for Digital and Material Technology of Stomatology, Beijing Key Laboratory of Oral Digital Medicine, Peking University School and Hospital of Stomatology, Beijing 100081, China.

**Keywords:** N-acetylcysteine, Gold nanoclusters, Acute kidney injury, Anti-inflammatory, Reactive oxygen species, Nanozyme

## Abstract

**Rationale:** Acute kidney injury (AKI) is associated with aberrant generation of oxidative species and inflammation, leading to high mortality of in-hospitalized patients. Although *N*-acetylcysteine (NAC) showed positive effects in alleviating contrast-induced AKI, the clinical applications are strongly restrained due to the low bioavailability, low renal accumulation, short renal retention time, and high dosage-induced toxicity.

**Methods:** We addressed the clinical dilemma of NAC by developing ultrasmall gold nanoclusters (1-2 nm) capped with NAC (denoted as Au NCs-NAC) as a nanozyme-based antioxidant defense system for AKI alleviation. Rhabdomyolysis-induced AKI mice model was developed, and the same dose of free NAC (as a control) and NAC onto Au NCs (Au NCs-NAC) was used for *in vivo* investigation of AKI restoration.

**Results:** The as-developed gold nanozyme exhibited high bioavailability and good physicochemical stability as compared to NAC. Meanwhile, Au NCs-NAC showed broad-spectrum antioxidant activity of Au NCs-NAC, offering *in vitro* renoprotective effects, as well as macrophages by relieving inflammation under hydrogen peroxide or lipopolysaccharide stimulation. Notably, owing to the smaller size than kidney threshold (5.5 nm), Au NCs-NAC displayed preferential renal enrichment (< 2 h) and longer retention (> 24 h) in AKI mice as revealed by fluorescence imaging, thereby largely enhancing the restoration of renal function in AKI mice than free NAC by protecting the kidneys from oxidative injury and inflammation without systemic toxicity, as demonstrated by tissues staining, inflammatory cytokines and biomarkers detection, and mice survival rate.

**Conclusion:** Owing to the synergistic anti-inflammatory/antioxidative effects, and enhanced bioavailability and renal accumulation/retention, Au NCs-NAC displayed far superior therapeutic performance than NAC alone. This work will facilitate the development of high-performance antioxidative nanoplatforms, as well as overcome the clinical limitations of small molecular drugs for AKI treatment and other inflammatory diseases.

## Introduction

Acute kidney injury (AKI), usually known as acute renal failure, is a devastating heterogeneous clinical renal disorder, greatly associated with high mortality and morbidity of critically ill in-hospitalized patients 1-3. Except renal replacement therapy and supportive care, no particular curative therapy is available yet towards this global health challenge 4,5. Pathologically, vascular damage, oxidative stress, and inflammation hold a prominent role in the onset and progression of AKI 6-12. Previously, small molecules such as *N*-acetylcysteine (NAC) and amifostine have been employed as antioxidants to restrict AKI progression due to their anti-inflammatory and antioxidative activities 13-15. Though NAC showed some success in relieving contrast-induced kidney damage, the low bioavailability, low renal accumulation, and short renal retention *in vivo* restrict its applications to relieve clinical AKI 16-18. In addition, the massive dose of NAC is usually required to achieve desired therapeutic efficacy, which may induce biotoxicity. Recently, our group demonstrated that the low dose (10 µg/mice) of NAC is unable to rescue renal function in rhabdomyolysis-induced acute kidney injury (RM-AKI) mice, whereas, on the other hand, though efficient renal protection was achieved at high dose (4.2 mg/mice) 19, the high dose still remains problematic regarding treatment safety 18-21. Therefore, different strategies are greatly demanded to overcome the clinical limitations of NAC and obtain effective therapeutic outcomes of AKI.

The advancements in nanotechnology shed light on AKI management 22-29. Particularly, the inorganic nanodelivery systems improve the bio-distribution and pharmacokinetics of drugs and facilitate targeted drug delivery to the specific organ 30-37. However, the delivery of drugs to renal tubules by nanodelivery systems is challenged by the glomerular filtration barrier (GFB) as most of nanocarriers possessed a large size than GFB (5.5 nm) and thus are non-specifically taken up by the mononuclear phagocyte system, leading to poor renal accumulation and high biotoxicity. Alternatively, ultrasmall nanozymes with high renal accumulation and both antioxidative and anti-inflammatory activities have arisen as promising candidates for AKI alleviation 38-42. Compared with small molecular drugs, these ultrasmall nanozymes with reasonable sizes can remain in the kidneys for a longer period and have better renal specificity. In previous reports, we demonstrated that ultrasmall nanomaterials with hydrodynamic diameter less than kidney threshold can effectively enrich in the kidneys, and can alleviate cisplatin as well as rhabdomyolysis-induced AKI owing to their multienzyme mimetic activity 43-46.

Recently, gold nanoclusters (Au NCs) (< 3 nm) have shown great promise due to the strong quantum confinement effect and physicochemical characterristics such as excellent biocompatibility, high colloidal stability, water-solubility, catalytic activity, and good photophysical property, and thus have been widely studied in drug delivery, biosensing, bioimaging, and disease treatment 47-50. Additionally, it has been reported that Au NCs possess multienzyme mimetic activity, thereby can effectively eliminate multiple reactive oxygen species (ROS), including hydroxyl radicals (·OH), superoxide anion, *etc.* 51-54. However, the direct antioxidant and anti-inflammatory activities of Au NCs towards AKI treatment have barely been explored. Hence, considering the potential risk of *in vivo* toxicity caused by gold, natural antioxidant-based ultrasmall gold nanozyme with both antioxidant and anti-inflammatory activities, preferential renal targeting, and low systemic toxicity, would be a potential candidate to alleviate AKI.

**Scheme 1:**
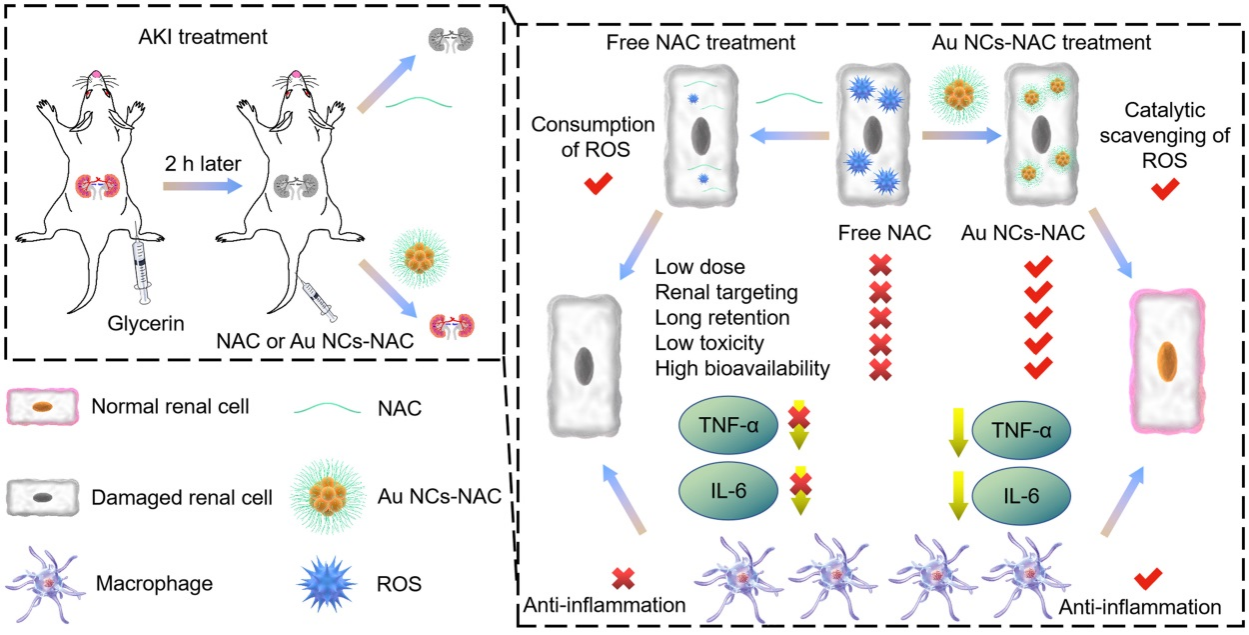
Schematic illustration of broad spectrum antioxidant and anti-inflammatory activities of ultrasmall Au NCs-NAC for AKI alleviation as compared with NAC.

Herein, antioxidant NAC was used both as a reductant and capping agent for the fabrication of ultrasmall gold nanoclusters as multienzyme mimetic nanozyme, offering effective AKI alleviation by the targeted clearance of ROS in the kidneys and the reduction of pro-inflammatory response. The as-developed Au NCs-NAC was 1-2 nm in diameter, and exhibited good water solubility and stability. In addition, owing to the multienzyme mimetic activity, Au NCs-NAC efficiently scavenged multiple ROS than NAC alone, leading to remarkable renal cells protection *in vitro* with negligible toxicity. Besides, ultrasmall Au NCs-NAC showed good bioavailability as inductively coupled plasma mass spectrometry (ICP-MS) and fluorescence imaging revealed their high renal enrichment *in vivo* upon intravenous administration. In comparison to NAC alone, Au NCs-NAC exhibited more excellent therapeutic effects in AKI mice *in vivo* at the same dose by scavenging ROS as well as reducing the release of inflammatory cytokines (interleukin-6, (IL-6), tumor necrosis factor-α (TNF-α)), respectively. Impressively, the renal function was protected and remarkably ameliorated in AKI mice as demonstrated by renal function indicators (creatinine (CRE) and blood urea nitrogen (BUN)), pro-inflammatory cytokines, hematoxylin-eosin (H&E) staining, and the biomarkers of kidney tissues. Consequently, broad spectrum oxidative stress and inflammatory regulation by Au NCs-NAC are demonstrated to relieve AKI with significantly prolonged survival rate of RM-AKI mice than free NAC. In comparison to free NAC from a clinical perspective, Au NCs-NAC hold great potential to treat clinical AKI. For instance, considering the high dosage-induced toxicity and low bioavailability of free NAC, Au NCs-NAC restored the clinical symptoms of AKI at low dose of NAC, exhibited superior renal targeting and long renal retention up to 24 h, better *in vivo* biocompatiblity, low toxicity, high bioavailability, and inflammatory regulation (**Scheme 1**). These features made Au NCs-NAC a promising clinical nanoagent for ameliorating AKI in clinic. Our work offers a new avenue to develop small molecular drug grafted ultrasmall nanozyme for the clinical management of AKI with high biosafety.

## Material and methods

### Materials

Chloroaurate hydrate (99.9%, metal basis) was obtained from Alfa Aesar (USA). Catalase from bovine liver (2000-5000 U/mg), 2,2′-azino-bis(3-ethylbenzothiazoline 6-sulfonate (ABTS), phosphate buffer saline (PBS), dulbecco's modified eagle medium (DMEM), fetal bovine serum (FBS), 2′,7′-dichlorofluorescein diacetate (DCFH-DA), 3-(4,5-dimethylthiazol-2-yl)-2,5-diphenyltetrazolium bromide (MTT), and lipopolysaccharide (LPS) were purchased from Sigma-Aldrich (USA). NAC and 3,3′,5,5′-tetramethylbenzidine (TMB) were obtained from Aladdin Reagent (China). Hydrogen peroxide (H_2_O_2_, 30%), hydrochloric acid (36%), and nitric acid were purchased from Guangzhou Chemical Reagent Factory, and used as received. Mito-Tracker Green assay kit was obtained from Beyotime Institute of Biotechnology. The TNF-α and IL-6 enzyme-linked immunosorbent assay (ELISA) assay kits were bought from ABBkine Scientific Company (USA). All chemicals and solvents were used without further purification. Deionized water, purified by a Milli-Q water purification system (Millipore, USA) to a minimum resistivity of 18.2 MΩ/cm, was used throughout the experiments.

### Characterizations

The sample morphology was characterized by a transmission electron microscopy (TEM, FEI Tecnai G2 Spirit, Holland). The morphology and size of sample were characterized by atomic force microscopy (AFM, Dimension ICON, Bruker, USA). X-ray photoelectron spectra (XPS) were acquired by using SSI S-Probe XPS spectrometer with Al Kα radiation as an X-ray source (1486 eV). The hydrodynamic size was measured by dynamic light scattering (DLS, 90Plus/BI-MAS instrument, Brookhaven Instruments Co., USA). The Fourier transform infrared (FT-IR) spectra were characterized by infrared spectrometer (L16000300 Spectrum TWO LITA, Llantrisant, UK) between 4000 and 500 cm^-l^ through a potassium bromide (KBr) pellet method. Thermogravimetric analysis (TGA) was performed using a Discovery TGA 50 (USA) instrument by scanning from 25 to 600 °C under oxygen at a heating rate of 10 °C/min. The UV-Vis absorption spectra were recorded by a UV-Vis-NIR spectrophotometer (Cary 60, Agilent Technologies, USA). The concentration of Au was measured using a Thermo inductively coupled plasma mass spectrometry (ICP-MS) XSERIES 2. Samples for ICP-MS analysis were prepared by dissolving Au NCs-NAC in aqua regia for overnight and diluted with ultrapure water. The *in vitro* ROS detection was performed by confocal laser microscopy (LSM880, Carl Zeiss, Göttingen, Germany).

### Synthesis of Au NCs-NAC

The Au NCs-NAC was synthesized according to the previous report with slight modifications 55,56. Chloroauric acid (2 mL, 20 mg/mL) and NaOH (3 mL, 0.5 M) were added in 20 mL NAC aqueous solution (80 mM), and stirred for 2.5 h at 37 °C. Then, the above aqueous solution was dialyzed (Mw cutoff: 2 kDa) for two days and the water was changed every 4 h. Finally, the resultant solution was stored in a refrigerator at 4 °C. The Au NCs without NAC as a control were also synthesized by the same method.

### Dark cytotoxicity, ROS detection, and anti-inflammation *in vitro*

For dark cytotoxicity *in vitro*, human embryonic kidney (HEK) 293T cells were incubated with various concentrations of Au NCs-NAC for 24 h and the cell viability was detected by the standard MTT assay. For the drugs-mediated protection of H_2_O_2_/LPS (H_2_O_2_: 0.5 mM; LPS: 400 ng/mL) stimulated cells experiment, the HEK 293T cells were pretreated with either Au NCs-NAC or NAC for 4 h, followed by H_2_O_2_/LPS treatment, and then the subsequent cell survival rate was determined by the standard MTT assay.

For ROS detection *in vitro*, HEK 293T cells were seeded in confocal dishes and incubated with/without NAC or Au NCs-NAC for 4 h. The cells were washed twice by PBS and stained with DCFH-DA or Mito-tracker in PBS for 15 min at 37 °C. Then, the cells were washed several times by PBS and imaged using a confocal laser microscopy after treated with 0.5 mM H_2_O_2_ for 24 h.

For anti-inflammation *in vitro*, both the macrophages and HEK 293T cells were seeded into 96-wells plates. Then, the HEK 293T cells were treated with LPS (400 ng/mL) in culture medium for 1 h. Next, the supernatant of HEK 293T cells were transferred into a 96-well plate to culture RAW264.7 macrophages for overnight. Finally, the concentrations of TNF-α and IL-6 in RAW264.7 macrophages supernatant were measured using TNF-α/IL-6 ELISA assay kit.

### Biodistribution of Au NCs-NAC* in vivo*

For fluorescence imaging, Au NCs-NAC was first labeled with IR-800. Both the healthy and AKI mice received an intravenous injection of IR-800 labeled Au NCs-NAC (2 mg/mL, 100 μL), and then imaged at different time points by using a small animal imager.

For ICP-MS analysis, the healthy and AKI mice (n = 3) were injected intravenously with Au NCs-NAC (2 mg/mL, 100 μL). Then, the mice were sacrificed to collect the main organs, which were then digested with aqua regia. The amount of Au in the major organs of the mice was detected by ICP-MS, and the ratio of injected Au in different organs to each gram of tissue was calculated.

### *In vivo* biosafety evaluation of Au NCs-NAC

Mice were intravenously administered with either PBS (100 μL) or Au NCs-NAC (2 mg/mL, 100 μL) (n = 3 per group), and their body weight was recorded for 30 days. All mice were euthanized on 30th day, and the serum biochemistry (BUN, CRE, alanine aminotransferase (ALT), and aspartate aminotransferase (AST)) and complete blood panel (white blood cells (WBC), lymphocyte (LYM), monocytes (MON), hematocrit (HCT), mean corpuscular volume (MCV), platelet distribution width (PDW), red blood cells (RBC), mean platelet volume (MPV), mean corpuscular hemoglobin (MCH), hemoglobin (HGB), mean corpuscular hemoglobin concentration (MCHC), and platelets (PLT)) were studied. Meanwhile, the major organs (heart, liver, spleen, lung, kidneys) were collected for H&E staining.

### *In vivo* AKI treatment

Twenty BALB/c mice were randomly assigned to five groups (n = 5): i. normal mice group (100 μL PBS); ii. Au NCs-NAC group (200 μg Au NCs-NAC in 100 μL PBS); iii. AKI mice PBS group (100 μL PBS); iv. NAC group (200 μg NAC in 100 μL of PBS) for AKI mice; v. Au NCs-NAC group (200 μg Au NCs-NAC in 100 μL of PBS) for AKI mice. In AKI mice model, NAC or Au NCs-NAC was administered intravenously at 2 h after intramuscular injection of 50% glycerol. Mice were euthanized at 24 h post-injection, and the renal function and inflammation were evaluated by comparing with normal mice. The body weights and survival curves of AKI mice were monitored for 14 days. According to the animal welfare guidelines, mice that lost more than 15 percent of their body weight were considered dead on the survival curve.

The serum of the euthanized mice was collected to determine the level of BUN and CRE. Meanwhile, the various organs from different groups were collected and fixed with 4% paraformaldehyde. Then, the samples were analyzed by H&E and immunohistochemical staining, respectively.

### Detection of biomarkers *in vivo*

The supernatant of homogenized renal tissues were stored in a refrigerator at -80 °C and tested according to the different experimental requirements. The level of lipid peroxidation was measured using a thiobarbituric acid reactive substances (TBARS) assay kit (Cayman Chemical, USA). The concentration of superoxide dismutase (SOD) was determined with a SOD assay kit (Sigma-Aldrich, USA). Kidney injury molecule-1 (KIM-1) and heme oxygenase-1 (HO-1) expression were evaluated using KIM-1 and HO-1 ELISA kit (Abcam, USA). The levels of TNF-α and IL-6 were determined by commercial TNF-α and IL-6 ELISA assay kits.

### Statistical analysis

The obtained experimental data were presented as means ± standard deviation. All statistical analyses were performed by Origin 8.5 software through One-Sample t-Test (ns: non-significant, *p < 0.05, **p < 0.01, ***p < 0.001).

## Results and Discussion

**Figure 1:**
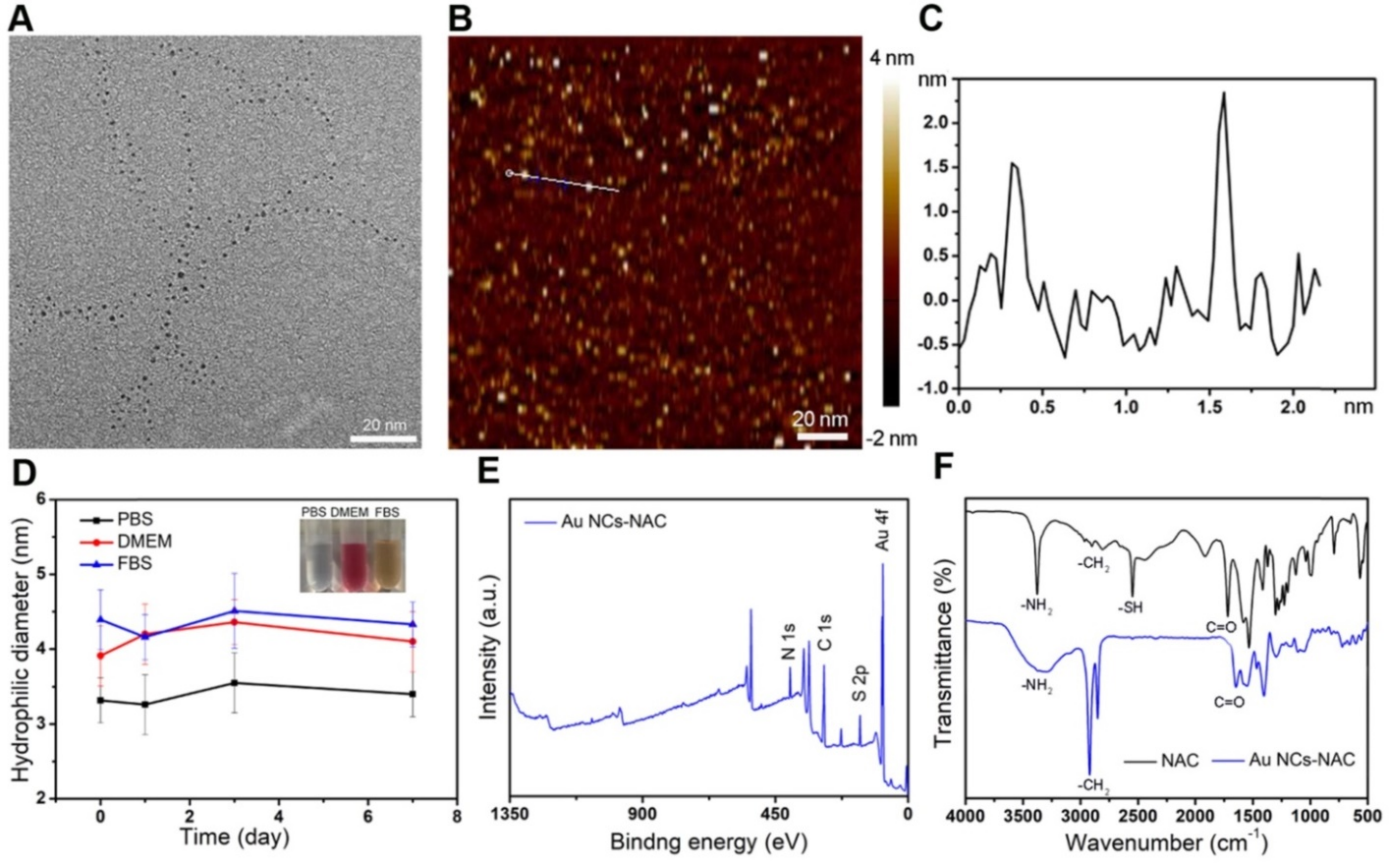
** (A)** HR-TEM image of Au NCs-NAC. **(B)** AFM image of Au NCs-NAC and **(C)** the height profile of Au NCs-NAC. **(D)** Hydrophilic diameter of Au NCs-NAC in PBS, DMEM, and FBS for 7 days. The inset is the digital photos of Au NCs-NAC dispersion in PBS, DMEM, and FBS. **(E)** XPS spectrum of Au NCs-NAC as indicated. **(F)** FTIR spectra of NAC and Au NCs-NAC.

In this work, Au NCs-NAC was obtained by a simple water phase synthesis method, following reported literature with some modifications 55,56. High-resolution transmission electron microscope (HR-TEM) characterization suggested the uniform distribution of Au NCs-NAC, with an average diameter of about 2 nm (**Figure 1 and S1**). As a control, Au NCs (~ 50 nm) without NAC were also prepared following the same protocol, which were characterized by TEM and DLS (Supporting Information **Figure S2A-B**). AFM image further confirmed that the as-obtained Au NCs-NAC is 2 nm in diameter and ~1-3 nm in thickness, respectively (**Figure 1B-C**). Notably, after incubation in different physiological solutions such as PBS, DMEM, and FBS for 7 days, an average hydrodynamic diameter of Au NCs-NAC was about 3-4 nm as determined by DLS (**Figure 1D**), which is even smaller than the GFB (5.5 nm), allowing an effective renal enrichment 57-60. Meanwhile, the zeta potential of Au NCs-NAC was measured as -47.8 ± 3.5 mV in **Figure S3** (Supporting Information). As shown in **Figure S4** (Supporting Information), Au NCs-NAC did not exhibit characteristic surface plasmon resonance peak (~520 nm), while under ultraviolet irradiation, they emitted strong red fluorescence at 650 nm, indicating the successful formation of Au NCs (Supporting Information **Figure S5**). In addition, the XPS spectra confirmed the presence of the binding energy of Au 4f (eV), C 1s (eV), N 1s (eV), and S 2p (eV) (**Figure 1E and S6**), implying the existence of both Au element and NAC. The surface properties of Au NCs-NAC were further studied by FTIR spectroscopy. **Figure 1F** displayed the FTIR spectra of both Au NCs-NAC and NAC. The peak corresponds to the S-H stretching vibration mode at 2547 cm^-1^ disappeared in Au NCs-NAC, confirming that NAC molecules are anchored onto the surface of Au NCs through Au-S bonding. In addition, TGA curve revealed ~ 50 wt% NAC onto Au NCs (Supporting Information **Figure S7**).

**Figure 2:**
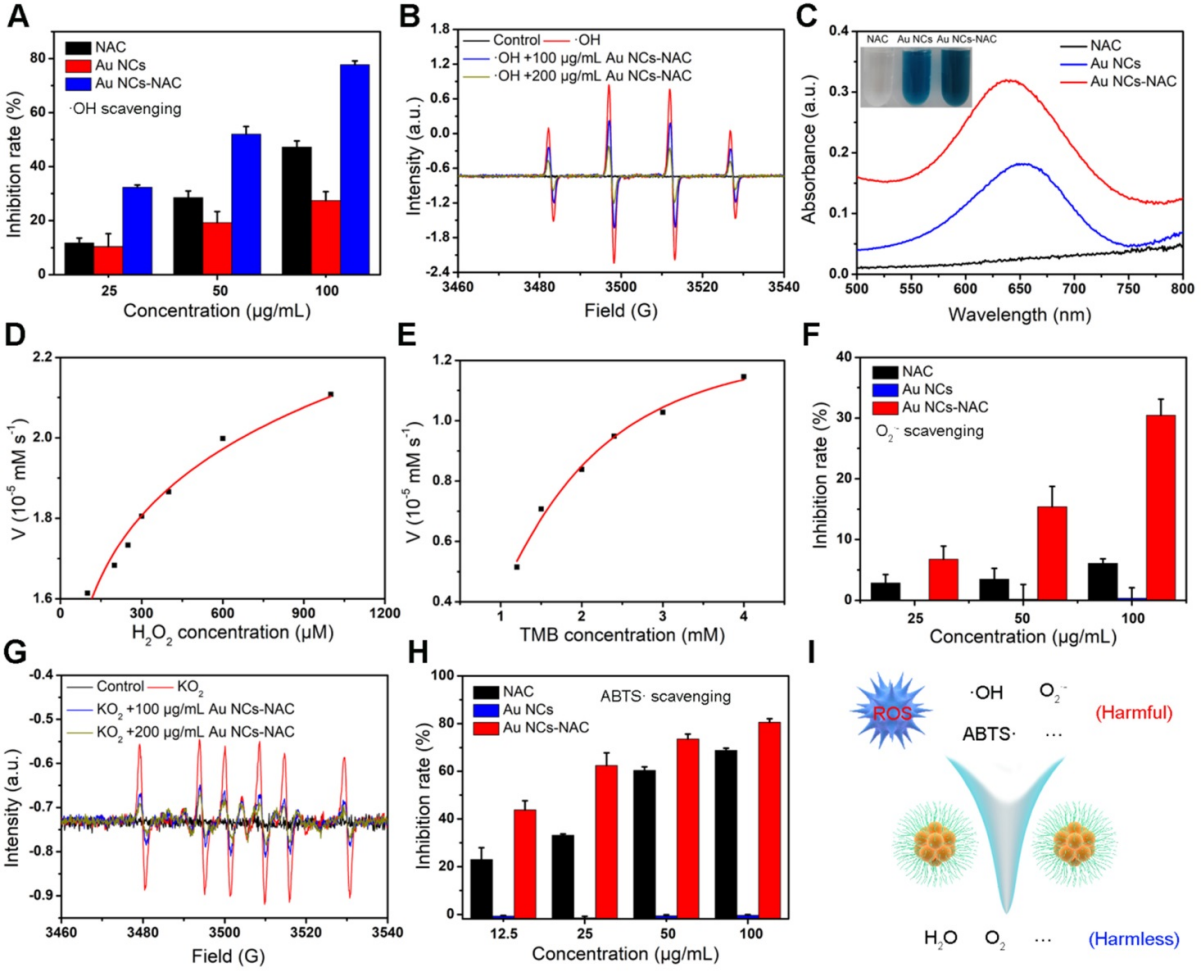
** (A)**·OH scavenging capacity of NAC and Au NCs-NAC. **(B)** ESR spectra of ·OH/DMPO adduct treated with/without Au NCs-NAC. **(C)** POD-like activity of NAC, Au NCs and Au NCs-NAC assessed by TMB assay. The inset is NAC, Au NCs and Au NCs-NAC solution containing H_2_O_2_ and TMB. Kinetic experiments of Au NCs-NAC with POD-like activity at varying concentrations of H_2_O_2_ **(D)** and TMB **(E)**. **(F)** O_2_·- radical scavenging activity of NAC and Au NCs-NAC. **(G)** ESR spectra of O_2_·-/DMPO adduct treated with/without Au NCs-NAC. **(H)** ABTS radical scavenging activity of NAC and Au NCs-NAC. **(I)** Illustration of the ROS-scavenging by Au NCs-NAC through catalytic conversion of harmful ROS (OH, O_2_·-, etc.) into harmless molecules such as H_2_O and O_2_, respectively.

Some natural enzymes like SOD, catalase (CAT), and glutathione peroxidase (GPx) can scavenge harmful ROS to maintain redox homeostasis *in vivo* 61-63. Au NCs have been reported to hold multiple enzyme-like activities, *e.g*., peroxidase (POD), SOD, and CAT, and thus widely used in different biomedical applications 52-55. First, the·OH scavenging ability of Au NCs-NAC was assessed. As presented in **Figure 2A**, Au NCs-NAC exhibit a concentration-dependent ·OH scavenging, which is far superior over free NAC. Approximately, 78% ·OH radicals were consumed by Au NCs-NAC at 100 μg/mL. A noticeable decrease in the characteristic peak of 5,5-dimethyl-1-pyrroline N-oxide (DMPO) and ·OH adduct also indicated that Au NCs-NAC could scavenge ·OH (**Figure 2B**). Subsequently, TMB color assay was used to assess the POD mimic activity of Au NCs-NAC and Au NCs in PBS solution at 25 °C. Compared to free NAC, Au NCs-NAC and Au NCs triggered an effective TMB oxidation in a time-dependent manner (**Figure 2C**). The K_m_ value of Au NCs-NAC as calculated by Michaelis-Menten equation was 0.2 and 4807.9 mM for TMB and H_2_O_2_, respectively (**Figure 2D**-**E**). These results demonstrated the better ROS scavenging ability of Au NCs-NAC than NAC, which is attributed to the intrinsic catalytic capacity of Au NCs. Furthermore, the SOD-like activity of Au NCs-NAC was estimated by a SOD assay kit. At 100 µg/mL concentration, Au NCs-NAC presented profound scavenging of O_2_·- (30.5 ± 2.6%) (**Figure 2F**), which is much higher than free NAC (6.1 ± 0.7%) and Au NCs (0.4 ± 1.7%). In addition, an obvious decrease was observed in the electron spin resonance (ESR) signal of O_2_·- and DMPO adduct upon the addition of Au NCs-NAC, which further confirmed an efficient elimination of O_2_·- by Au NCs-NAC (**Figure 2G**). Finally, ABTS free radical (ABTS·) assay was used to determine the broad-spectrum free radical scavenging capacity of Au NCs-NAC (**Figure 2H**). A concentration-dependent scavenging behavior was also noticed towards ABTS· as 100 µg/mL Au NCs-NAC exhibited higher ABTS· scavenging (80.6 ± 1.5%) than NAC (68.9 ± 0.9%) and Au NCs (-0.3 ± 0.3%). The ROS scavenging ability of Au NCs-NAC is superior to Au NCs, which is ascribed to the presence of NAC ligand, improving the overall enzyme-like activity of Au NCs-NAC 64,65. Meanwhile, the results of electrospray ionization-mass spectrometry (ESI-MS) showed that a part of NAC on Au NCs assisted the removal of ROS and produce reduced NAC (Supporting Information **Figure S8**). Furthermore, cytochrome c (Cyt c) assay was performed to explore the mechanism of the multi-enzyme mimetic feature of Au NCs-NAC. Without Au NCs-NAC, a characteristic spectrum of Cyt c was observed with a prominent absorption in a visible region (520 nm and 550 nm) as shown in **Figure S9**, indicating the reduced state of Cyt c. However, a new peak at 530 nm was found by Cyt c-treated with Au NCs-NAC either in air or N_2_ inert environment, without the two characteristic absorption peaks of Cyt c. These results demonstrated that regardless of the dissolved oxygen, Au NCs-NAC can accept electrons from Cyt c. All results verified the multi-enzyme mimetic feature of Au NCs-NAC with superior ROS scavenging activity, suggesting enormous potential as a promising antioxidant for AKI therapy. **Figure 2I** shows the transformation of harmful ROS into harmless product by Au NCs-NAC.

**Figure 3:**
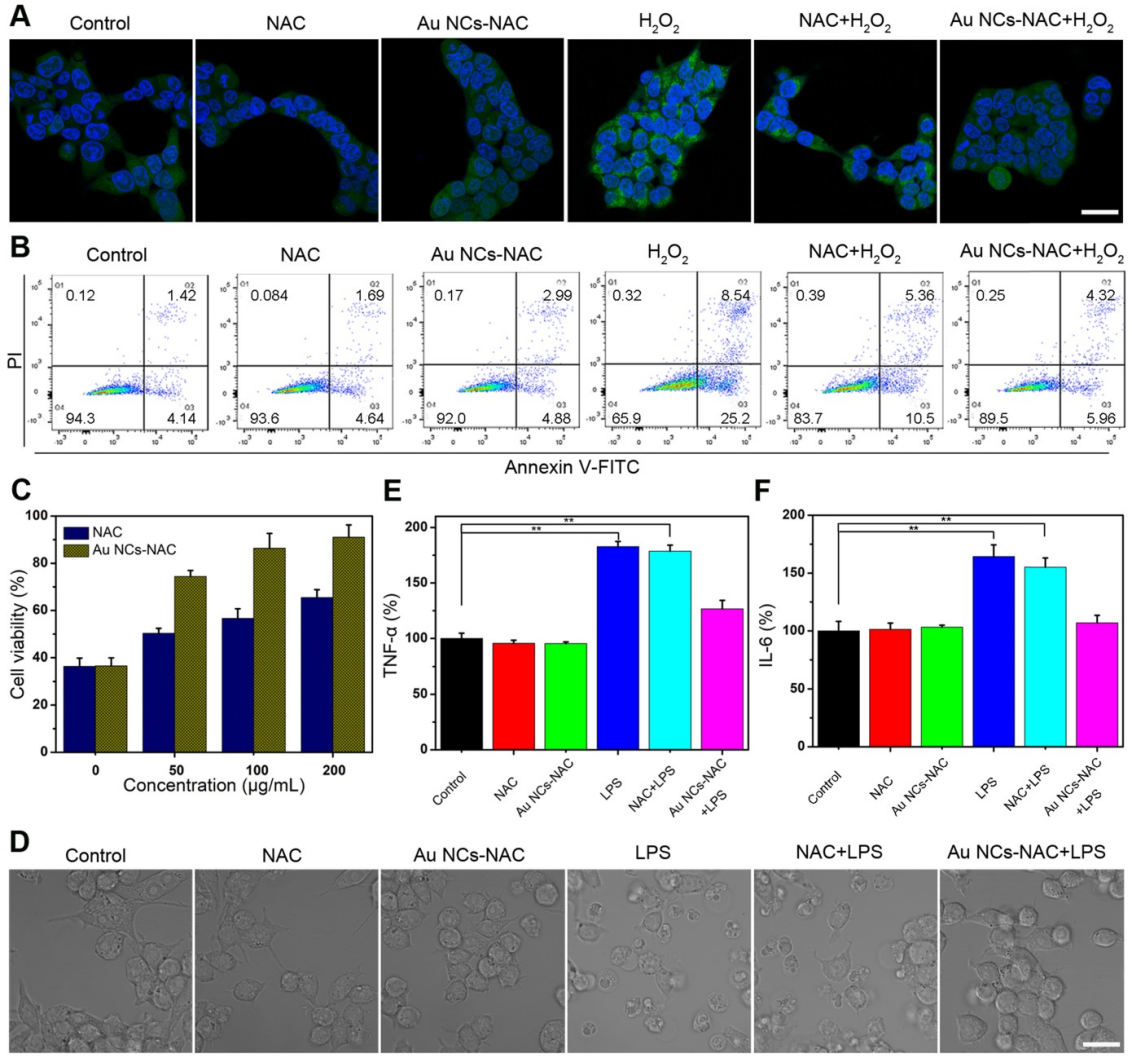
** (A)** ROS level in HEK 293T cells stained with DCF under different conditions. The scale bar is 20 µm. **(B)** PI/Annexin V-FITC staining of HEK 293T cells under different conditions. **(C)** Cell viability of HEK 293T cells stimulated by H_2_O_2_ and pre-treated with NAC or Au NCs-NAC. **(D)** Confocal images of macrophages after different treatments. The level of TNF-α **(E)** and IL-6 **(F)** in culture medium incubated with/without LPS and pre-treated with NAC or Au NCs-NAC. The scale bar is 20 µm. Data represent the mean ± S.D (n = 3). **P < 0.01 versus control group, respectively.

First, the intracellular uptake of Au NCs-NAC was measured by ICP-MS. We found that the intracellular uptake of Au NCs-NAC increases with an increase in incubation time as the content of Au in HEK 293T cells was about 695, 1210, and 1365 ng/10^6^ cells at 2, 4, and 8 h, respectively (Supporting Information **Figure S10**). The *in vitro* dark cytotoxicity of Au NCs-NAC was then investigated by a standard MTT assay. **Figure S11** indicated more than 80% survival of HEK 293T cells, which suggests no significant toxicity of Au NCs-NAC in dark. H_2_O_2_, one of the common intracellular ROS, was added externally to increase the level of intracellular ROS. After H_2_O_2_ treatment, an intense fluorescence signal was recorded in HEK 293T cells stained by ROS probe DCFH-DA, while Au NCs-pretreated cells exhibit significantly decreased fluorescence, which was close to the normal level (**Figure 3A**). Mitochondria, which is known as a power house of cells, is more vulnerable to ROS damage, and thus stained by mitochondrial probe Mito-tracker to determine ROS-induced damage. As shown in **Figure S12**, intracellular mitochondrial structure is destroyed after H_2_O_2_ stimulation, whereas, the pretreatment with Au NCs-NAC significantly improved the mitochondrial integrity of the cells. Considering that AKI is associated with severe apoptosis of renal cells, an apparent apoptosis (> 33%) was noticed in HEK 293T cells (**Figure 3B and S13**), while the cells pretreated with Au NCs-NAC present significantly decreased apoptosis rate (10.3%), which is even lower than free NAC (15.9%). Moreover, an enhanced survival rate of H_2_O_2_-stimulated HEK 293T cells was recorded after pretreatment with Au NCs-NAC (**Figure 3C**), confirming an efficient protection of renal cells by Au NCs-NAC through ROS elimination. On the other hand, AKI led to induce strong inflammatory responses, which trigger macrophages to release inflammatory factors, such as TNF-α and IL-6, respectively. LPS, which is derived from the outer membrane of Gram-negative bacteria, is commonly employed as an inflammation inducer 66,67. As displayed in **Figure 3D**, LPS stimulation (400 ng/mL) changed the morphology of RAW264.7 cells, however, upon the addition of both Au NCs-NAC and LPS, no particular morphological change was seen, which is even comparable to the control group, further confirming the ROS elimination to avoid the activation of macrophages. Moreover, the supernatant of RAW264.7 macrophages was used to determine the level of TNF-α and IL-6. Compared to the control group, 2.5- and 1.8-fold higher IL-6 and TNF-α contents were recorded after LPS treatment alone, suggesting ROS-induced inflammation (**Figure 3E-F**). Whereas, a profound decrease in IL-6 (1.5-fold) and TNF-α (1.2-fold) levels was noticed upon both Au NCs-NAC and LPS treatment, respectively. Importantly, the cellular viability of LPS-treated cells was also significantly improved after pre-incubation with Au NCs-NAC (**Figure S14**). It is notable to mention that free NAC at low dose cannot provide sufficient protection to renal cells from oxidative stress and inflammation as verified by *in vitro* experiments, whereas Au NCs-NAC displayed efficient renoprotective effects *in vitro* through ROS scavenging as well as anti-inflammatory effects, respectively.

**Figure 4:**
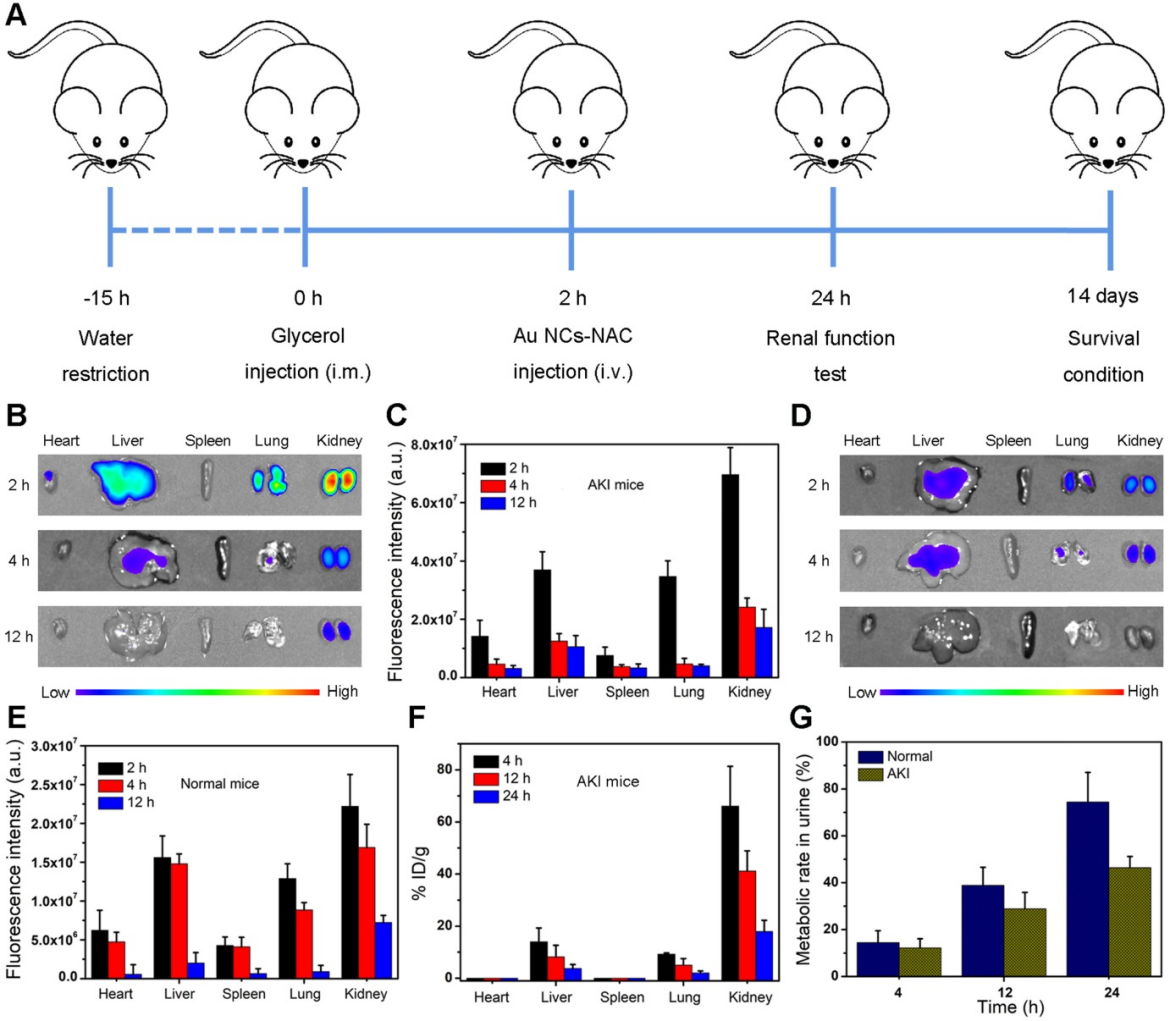
** (A)** Construction and treatment evaluation schedule of AKI model mice. **(B)** Ex-vivo fluorescence imaging and **(C)** the quantitative analysis of Au NCs-NAC in the major organs of AKI mice. **(D)** Ex-vivo fluorescence imaging and **(E)** the quantitative analysis of Au NCs-NAC in the major organs of normal mice. **(F)** Au content in the major organs of AKI mice after *i.v.* injection of Au NCs-NAC at indicated time points. **(G)** Au content in the urine of AKI mice after *i.v.* injection of Au NCs-NAC at indicated time points.

Fluorescence imaging and ICP-MS were employed to evaluate the biodistribution of Au NCs-NAC *in vivo*. First, RM-AKI mice model was established as shown in **Figure 4A**. Subsequently, IR800 dye was coupled with Au NCs-NAC through covalent bonding for *in vivo* fluorescence imaging. **Figure S15** showed negligible decrease in the fluorescence signal, indicating good stability of IR800-labeled Au NCs-NAC. Next, fluorescence images of the major organs collected from both the normal and AKI mice were captured at an indicated time points (2, 4, and 12 h) after systemic injection of IR800-labeled Au NCs-NAC. As shown in **Figure 4B-C**, the AKI mice treated with IR800-labeled Au NCs-NAC exhibited relatively higher fluorescence intensity in the kidneys at 2 and 4 h post-injection, indicating rapid renal accumulation of Au NCs-NAC. However, the normal mice showed much weaker fluorescence signals in the kidneys after intravenous (*i.v.*) administration (**Figure 4D-E**), which is mainly due to the normal metabolism of normal mice. Interestingly, the fluorescence signals in both the mice model were significantly weakened after 12 h post-injection, suggesting the rapid excretion of Au NCs-NAC from the body. The major organs and urine were also collected at different *i.v.* post-injection time points (4, 12, and 24 h) for ICP-MS analysis, which confirmed the preferential accumulation of Au NCs-NAC in the kidneys and rapid clearance *via* urine (**Figure 4F-G**). In particular, the maximum Au NCs-NAC (66% ID/g) were enriched in the kidneys at 4 h, and a very low amount of Au NCs-NAC was found in the urine (healthy: 14.4% and AKI: 12.2%). On the other hand, ~ 74.4% and 46.4% Au NCs-NAC were collected in the urine of healthy and AKI mice after 24 h post-*i.v.* injection, suggesting the renal excretion of Au NCs-NAC from mice. Such a preferential enrichment and quick body clearance are highly advantageous for AKI treatment, and is strongly ascribed to the ultrasmall size of the as-designed multi-enzyme mimetic Au NCs-NAC.

In contrast to AKI mice, the impact of intravenously injected Au NCs-NAC (10 mg/kg) onto the healthy mice was also studied for 1 month. *In vivo* biological safety was assessed by determining the blood biochemistry, major organs histopathology, and the body weight of mice. Major organs did not present any obvious damage or inflammation as shown in **Figure S16-S17**. Further, the serum biochemical analysis (Supporting Information **Figure S18A-B**) and complete blood panel (Supporting Information **Figure S19A-D**) displayed that hematological parameters and the concentrations of renal (BUN and CRE) and liver function indicators (aspartate aminotransferase (AST) and alanine aminotransferase (ALT)) had no noticeable differences between the PBS and Au NCs-treated mice, respectively. Importantly, no apparent change was noticed in the mice body weight after *i.v.* injection of Au NCs-NAC for 1 month (Supporting Information **Figure S20**). Meanwhile, the high concentration of Au NCs-NAC (2 mg/mL) has no obvious effect (hemolysis rate <5%) on red blood cells as shown in **Figure S21**, indicating the good biocompatibility of Au NCs-NAC. These results verified an excellent *in vivo* biocompatibility of Au NCs-NAC, indicating their potential for AKI treatment *in vivo* with negligible biotoxicity.

**Figure 5:**
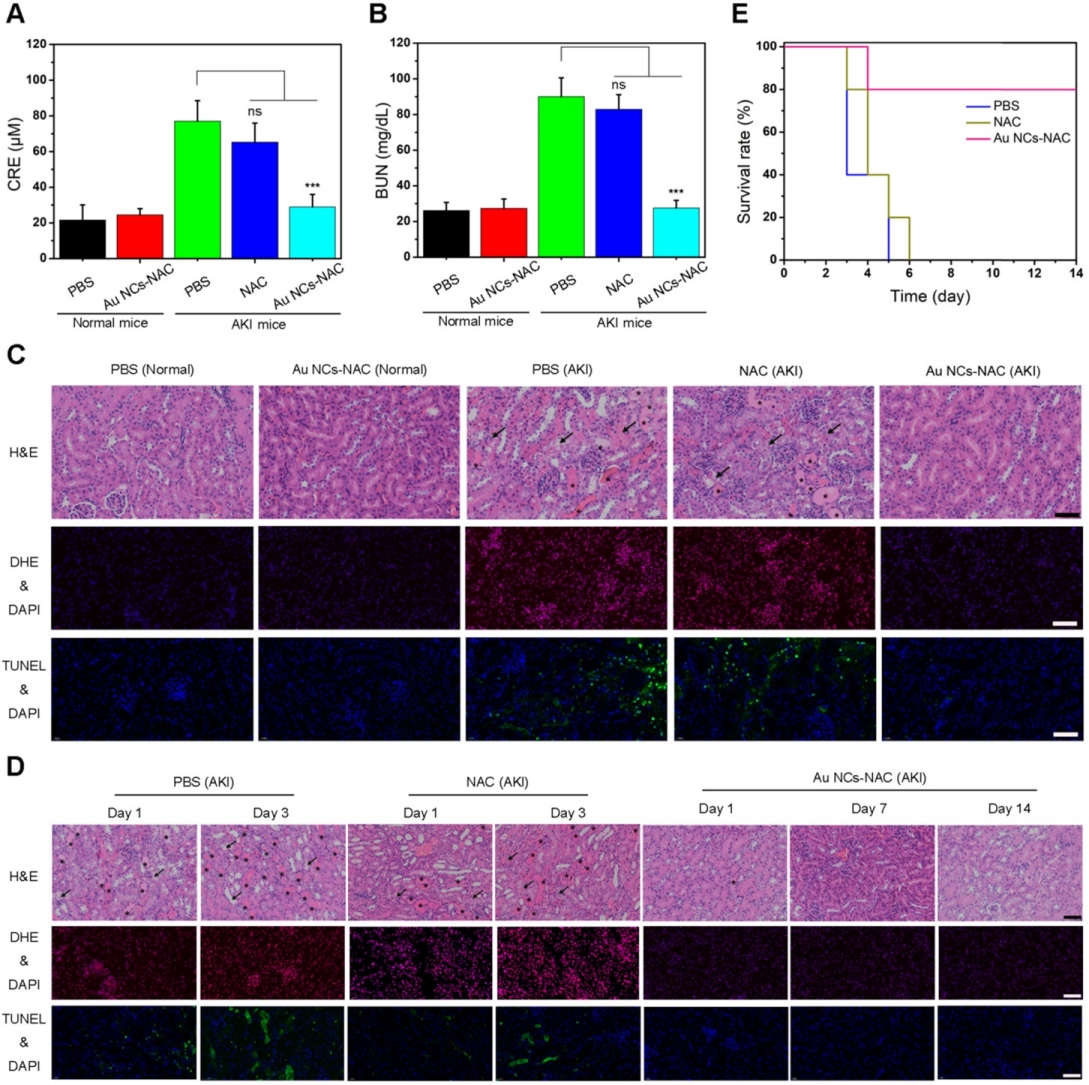
The levels of CRE **(A)** and **BUN (B)** in serum under different conditions. Data represent the mean ± S.D (n = 5). ***p < 0.001 and ns: non-significant versus AKI PBS-treated group, respectively. **(C)** DHE-stained fluorescence images, H&E-stained images and TUNEL-stained fluorescence images of renal tissues under different conditions. **(D)** DHE-stained fluorescence images, H&E-stained images and TUNEL-stained fluorescence images of renal tissues of AKI mice after treated with PBS, free NAC or Au NCs-NAC at indicated time points. The damaged tubules were marked as arrows and the formation of casts were marked as asterisks in (C) and (D). **(E)** Survival curves of AKI mice after treated with PBS, free NAC or Au NCs-NAC for 2 weeks. The scale bars are 100 μm in H&E-stained images, the scale bars are 50 μm in DHE-stained and TUNEL-stained fluorescence images.

First, the concentrations of two clinically important renal indicators (BUN and CRE) were determined after treatment for 24 h 19,68. The levels of BUN and CRE are much higher in AKI mice, while after *i.v.* administration of Au NCs-NAC, AKI mice exhibit a remarkable reduction in CRE and BUN level up to the normal mice (**Figure 5A-B**), suggesting the repairment and recovery of renal function. Meanwhile, H&E-stained renal tissues harvested from different treatment groups provided more direct evidence regarding the superior therapeutic effects of Au NCs-NAC. Many damaged tubules (marked as arrows) as well as the formation of casts (marked as asterisks) were seen in the kidney tissues of control group (**Figure 5C**). However, no damage structure was found in Au NCs-NAC-treated AKI mice. Given the fact that ROS-induced AKI often triggers cellular apoptosis, the ROS level and apoptotic cells in the kidney tissues were investigated by dihydroethidium (DHE) staining and terminal deoxynucleotidyl transferase-mediated deoxyuridine triphosphate nick end labeling (TUNEL) assay, respectively. Compared to the healthy mice, PBS-treated AKI group showed an obvious increase in ROS level and apoptotic cells, however, much reduced ROS level and apoptosis were noticed in Au NCs-NAC treated mice, indicating excellent ROS scavenging and anti-apoptotic effects of Au NCs-NAC (**Figure 5C**). Notably, Au NCs-NAC displayed significantly superior treatment outcomes at the same dose than free NAC. Moreover, after 14 days of treatment, the survival curve, body weight changes, H&E staining of renal tissues, renal indicators, ROS level, and apoptotic cells in renal tissues (**Figure 5D-E**, Supporting Information **Figure S22-S23**) further demonstrated the promising ability of Au NCs-NAC to alleviate AKI *in vivo*.

**Figure 6:**
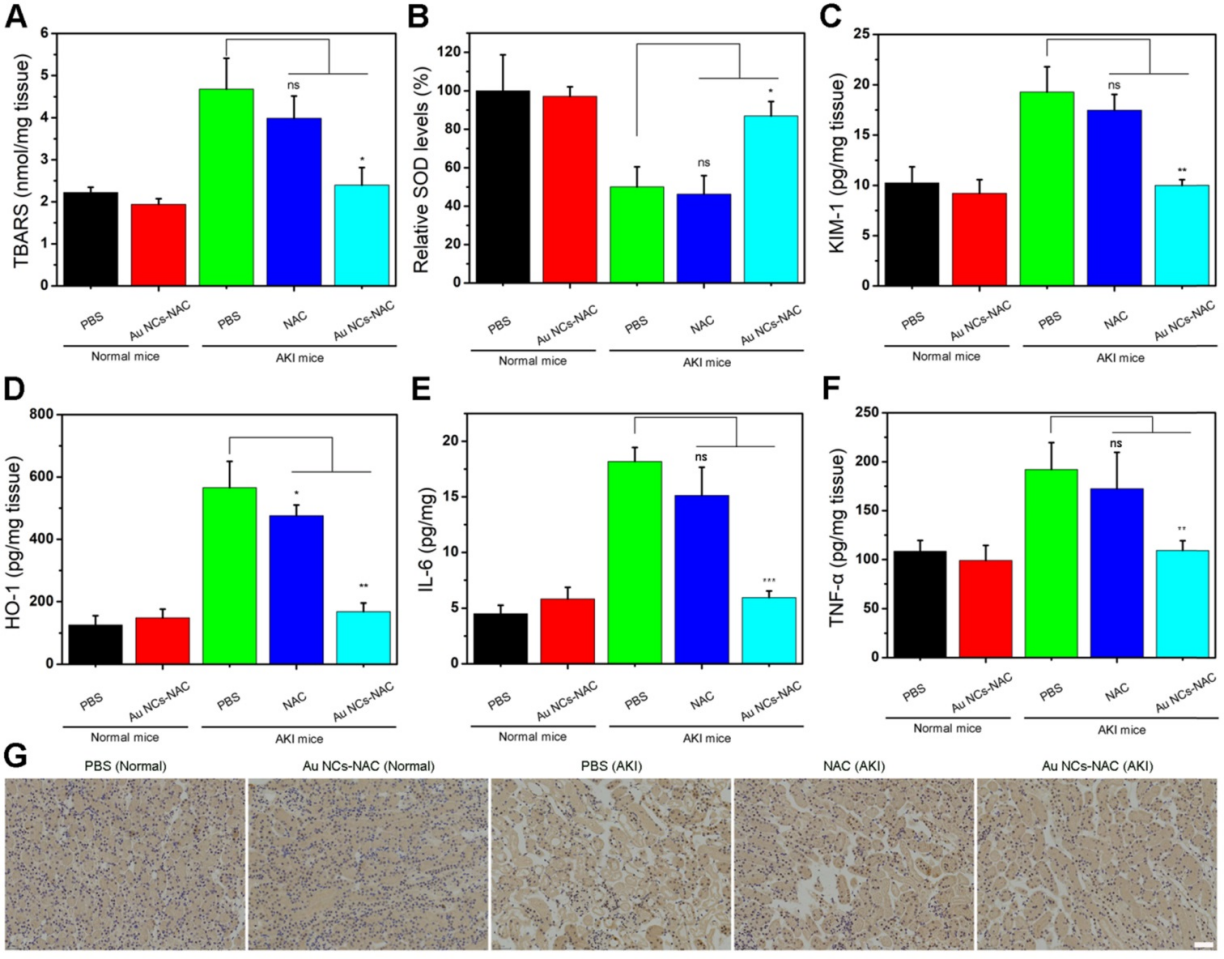
** (A)** Expression of TBARS in renal tissues under different conditions. **(B)** SOD level in renal tissues under indicated conditions. Expression of KIM-1 **(C)** and HO-1 (D) in renal tissues under the mentioned conditions. **(E)** IL-6 and **(F)** TNF-α levels in renal tissues under different treatment conditions. Data represent the mean ± S.D (n = 3). ***p < 0.001, **P < 0.01, *P < 0.05, and ns: non-significant versus AKI PBS-treated group, respectively. **(G)** Immunohistochemical staining images of CD68 in renal tissues under different conditions as indicated. The scale bar is 50 μm.

Excess ROS and inflammation usually cause lipid peroxidation in cells. Hence, the level of lipid peroxidation was further determined using a standard assay kit. **Figure 6A** presented a higher lipid peroxidation in the PBS-treated AKI group, while a significantly reduced lipid peroxidation up to the level of control group was noticed in Au NCs-NAC treated AKI mice group. Meanwhile, the SOD activity was much higher in the kidneys of both Au NCs-treated AKI mice than PBS-treated group (**Figure 6B**), indicating that Au NCs-NAC could protect kidneys by scavenging ROS and maintaining SOD activity. The level of two crucial kidney injury biomarkers such as kidney injury molecule-1 (KIM-1) and heme oxygenase-1 (HO-1) were also examined 69,70. Compared to healthy mice, a higher expression of KIM-1 and HO-1 was recorded in PBS-treated AKI mice, whereas a prompt drop in their expression level was seen in Au NCs-NAC treated AKI mice model (**Figure 6C-D**). Similarly, Au NCs-treated groups also presented an obvious reduction in pro-inflammatory factors (IL-6 and TNF-α) than PBS-treated AKI mice (**Figure 6E** and **F**), confirming that Au NCs-NAC effectively reduced the pro-inflammatory response in the kidneys. Moreover, there was an obvious increase in macrophages in the kidneys of AKI group. The NAC treatment did not reduce the AKI-induced macrophages infiltration, while fewer macrophages were observed in Au NCs-NAC treated group (**Figure 6G** and **S24**). These results explain the high effectiveness of Au NCs-NAC for AKI therapy.

## Conclusions

In this study, we have developed the renal targeted ultrasmall NAC-capped Au NCs as bimodal detoxification nanozyme for alleviating AKI. The as-obtained Au NCs-NAC exhibited broad spectrum antioxidant and anti-inflammatory activities. In addition, fluorescence imaging and ICP-MS revealed renal targeting of Au NCs-NAC with rapid clearance from the body, which further improves the therapeutic efficacy and biocompatibility of nanozymes. *In vitro* and *in vivo* experiments confirmed that Au NCs-NAC can protect renal cells by removing intracellular superfluous ROS and ROS-induced inflammation than free NAC. Moreover, blood biochemical analysis, pro-inflammatory cytokines detection, H&E staining analysis, biomarkers detection of kidney tissues, and mice survival rate demonstrated that Au NCs-NAC efficiently alleviate and treat AKI at low dose via robust antioxidative protection and anti-inflammation than free NAC. To conclude, the present work presented a promising strategy to enhance the therapeutic efficacy of small molecular antioxidants at low dose, which could be expanded to natural antioxidants to accelerate their clinical translation for AKI and other ROS-related diseases.

## Supplementary Material

Supplementary figures.

## Abbreviations

AKI: Acute kidney injury
NAC: *N*-acetylcysteine
gold nanoclusters: Au NCs
Au NCs-NAC: gold nanoclusters capped with NAC
RM-AKI: rhabdomyolysis-induced acute kidney injury
GFB: glomerular filtration barrier
ROS: reactive oxygen species
ICP-MS: inductively coupled plasma mass spectrometry
IL-6: interleukin-6
TNF-α: tumor necrosis factor-α
CRE: creatinine
BUN: blood urea nitrogen
H&E: hematoxylin-eosin
ABTS: 2,2′-azino-bis(3-ethylbenzothiazoline 6-sulfonate
PBS: phosphate buffer saline
DMEM: dulbecco's modified Eagle's medium
FBS: fetal bovine serum
DCFH-DA: 2′,7′-dichlorofluorescein diacetate
MTT: 3-(4,5-dimethylthiazol-2-yl)-2,5-diphenyltetrazolium bromide
LPS: Lipopolysaccharide
TMB: tetramethyl benzidine
H_2_O_2_: hydrogen peroxide
ELISA: enzyme-linked immunosorbent assay
TEM: transmission electron microscopy
AFM: atomic force microscopy
XPS: X-ray photoelectron
DLS: dynamic light scattering
FT-IR: Fourier transform infrared
TGA: thermogravimetric analysis
HEK: human embryonic kidney
WBC: white blood cells
LYM: lymphocyte
MON: monocytes
HCT: hematocrit
MCV: mean corpuscular volume
PDW: platelet distribution width
RBC: red blood cells
MPV: mean platelet volume
MCH: mean corpuscular hemoglobin
HGB: hemoglobin
MCHC: mean corpuscular hemoglobin concentration
PLT: platelets
AST: aspartate aminotransferase
ALT: alanine aminotransferase
SOD: superoxide dismutase
KIM-1: kidney injury molecule-1
HO-1: heme oxygenase-1
TBARS: Thiobarbituric acid reactive substances
HR-TEM: high-resolution transmission electron microscope
CAT: catalase
GPx: glutathione peroxidase
POD: peroxidase
·OH: hydroxyl radicals
DMPO: 5,5-dimethyl-1-pyrroline N-oxide
ABTS free radical: (ABTS·)
ESI-MS: electrospray ionization-mass spectrometry
Cyt c: cytochrome c
ESR: electron spin resonance
i.v.: intravenous
DHE: dihydroethidium
TUNEL: terminal deoxynucleotidyl transferase-mediated deoxyuridine triphosphate nick end labeling
