# Supplementary material for: Clinically translatable gold nanozymes with broad spectrum antioxidant and anti-inflammatory activity for alleviating acute kidney injury
Source: Theranostics. 2021 Oct 17;11(20):9904–17. doi: 10.7150/thno.66518 (PMC8581429; doi:10.7150/thno.66518)
Supplement: Supplementary file 1 — Supplementary figures. [file thnov11p9904s1.pdf]

Supporting Information

Clinically translatable gold nanozymes with broad spectrum antioxidant and anti-inflammatory activity for alleviating acute kidney injury

Dong-Yang Zhang[a,b,†], *Tianhui Tu*[a,†], *Muhammad Rizwan Younis*[a,b,†], *Kathy S. Zhu*[a,c], *Hengke Liu*[a], *Shan Lei*[a,b], *Junle Qu*[b], *Jing Lin*[a] *and Peng Huang*[a]*

[a]Marshall Laboratory of Biomedical Engineering, International Cancer Center, Laboratory of Evolutionary Theranostics (LET), School of Biomedical Engineering, Shenzhen University Health Science Center, Shenzhen 518060, China

[b]Key Laboratory of Optoelectronic Devices and Systems of Ministry of Education and Guangdong Province, College of Optoelectronic Engineering, Shenzhen University, Shenzhen 518060, China

[c]National Clinical Research Center for Oral Diseases, National Engineering Laboratory for Digital and Material Technology of Stomatology, Beijing Key Laboratory of Oral Digital Medicine, Peking University School and Hospital of Stomatology, Beijing 100081, China

Corresponding author:

Peng Huang

Addresses: 1066 Xueyuan Boulevard Shenzhen University Health Science Center, Shenzhen, 518060, China. E-mail: peng.huang@szu.edu.cn (Peng Huang)

[†]These authors contributed equally to this work.

Methods

POD-like activity of Au NCs-NAC

The POD-like activity of Au NCs-NAC was evaluated by 3,3',5,5'-tetramethyl-benzidine dihydrochloride (TMB) assay in the presence H_2O_2 in phosphatic buffer solution (pH 7.4) at room temperature. The absorbance of solution containing TMB (2 mM), H_2O_2 (1 M) and Au NCs-NAC (30 μg/mL) was recorded every 1 min till 5 min.

The kinetic experiments of Au NCs-NAC for POD-like activity was conducted at room temperature in phosphatic buffer solution (pH 7.4) with Au NCs-NAC (30 μg/mL) in the presence of H_2O_2 and TMB. The kinetic analysis of Au NCs-NAC with TMB as a substrate was performed by varying the concentrations of TMB at a fixed H_2O_2 concentration and vice versa. Finally, the catalytic parameters were calculated by fitting the Michaelis-Menten equation.

Hydroxyl radical (·OH) scavenging activity of Au NCs-NAC

The ·OH scavenging activity of Au NCs-NAC was evaluated using a radical antioxidant capacity (HORAC) assay kit (Cell Biolabs, Inc., USA). Experiments with different concentrations of Au NCs-NAC were carried out according to the instructions provided by the manufacturer. Further, 5 mM H_2O_2, 50 μM $FeCl_2$, and 100 μg/mL of Au NCs-NAC or free NAC were mixed in PBS (pH 7.4), and ESI-MS spectra were recorded.

ABTS radical (ABTS·) scavenging activity of Au NCs-NAC

The ABTS radicals (ABTS·) were first obtained by mixing 7 mM ABTS with 2.45 mM potassium persulfide for 12 h. Next, the absorbance of ABTS· solution with 12.5-100 μg/mL Au NCs-NAC (A_S) and pure ABTS· solution (A_A) was determined at 734 nm. The scavenging efficiency of Au NCs-NAC was calculated by the following formula: $[(A_A - A_S)/A_A] * 100$. All measurements were made in triplicate.

Superoxide anion radical ($O_2^{\cdot-}$) scavenging activity of Au NCs-NAC

The $O_2^{\cdot-}$ scavenging capacity of various concentrations of Au NCs-NAC was assessed by a SOD assay kit (Sigma-Aldrich, USA). The experiment was conducted in full accordance with the instructions provided by the supplier. The ESR signals of sample containing 100 mM DMPO, 100 μg/mL of Au NCs-NAC and 10 mM KO_2 were recorded.

Cytochrome c (Cyt c) electron transfer experiment

Au NCs-NAC (100 μg/mL) were mixed with Cyt c (400 μM) in PBS (pH = 7.4) in dark for 1 h. Then, UV-vis spectrophotometer was used to record the absorption of the resultant solution. To determine whether Cyt c was oxidized by the dissolved oxygen, N_2 gas was pumped for 1 h in the stock solutions of Cyt c, Au NCs-NAC solution, and PBS, simultaneously.

Subsequently, the experiment was repeated following the above-described process.

Hemolysis assay

For hemolysis assay, the whole blood was drawn from all mice and washed with PBS to obtain a suspension of red blood cells (RBC). Then, RBC suspension (0.2 mL) was mixed with different concentrations (0.2 mg/mL, 0.8 mL) of Au NCs-NAC for 6 h, followed by the centrifugation. Finally, the absorbance of a supernatant was measured at 541 nm. The hemolysis rate of Au NCs-NAC was calculated as follow: Hemolysis (%) = $(A_s - A_{PBS})/(A_{water} - A_{PBS}) \times 100\%$, where A_s, A_{PBS}, and A_{water} are the absorbance of the sample groups, the PBS group, and the water group, respectively.

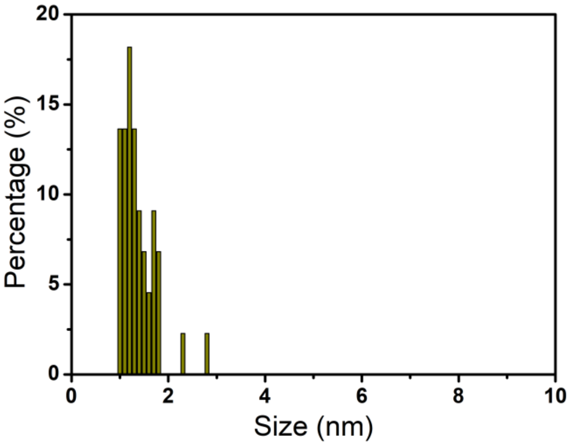

Figure S1. The size distribution of Au NCs-NAC was statistically analyzed by HR-TEM image.

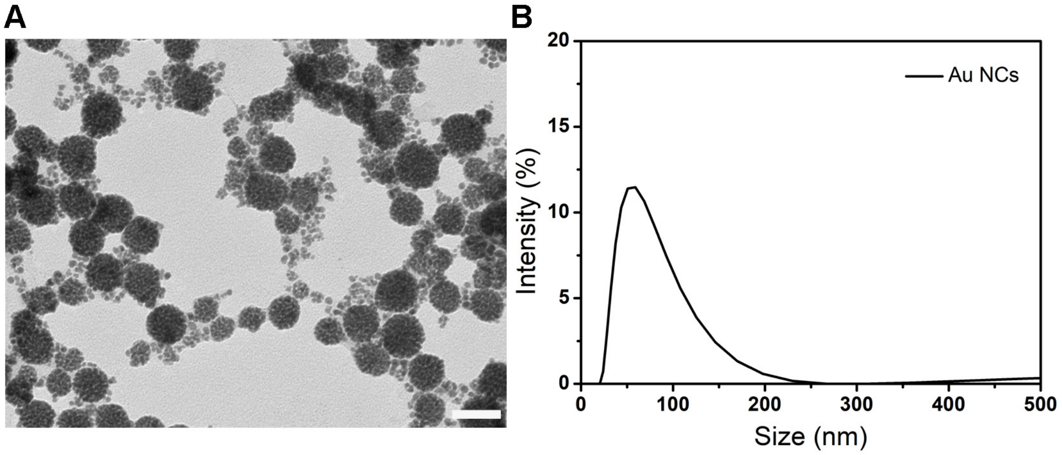

Figure S2. (A) TEM image of Au NCs. The scale bar is 50 nm. (B) Hydrophilic diameter of Au NCs as determined by DLS.

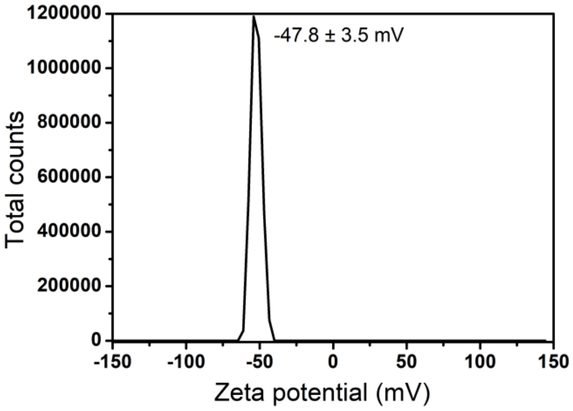

Figure S3. The zeta potential of Au NCs-NAC.

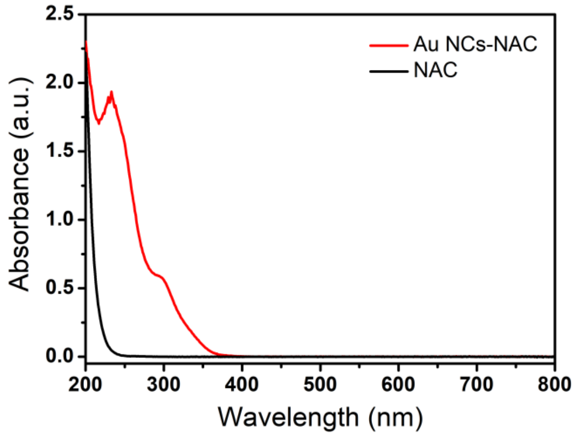

Figure S4. Absorption spectra of Au NCs-NAC and NAC.

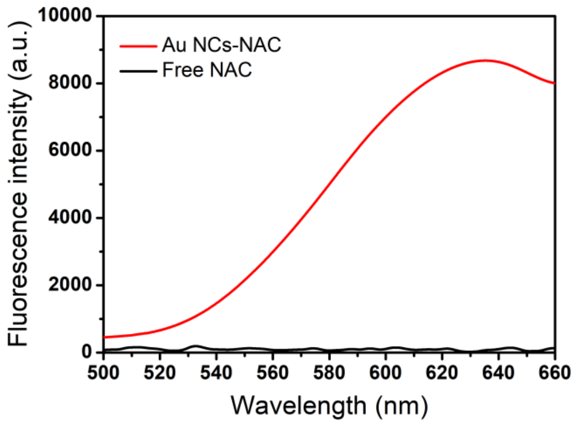

Figure S5. Fluorescence spectra of Au NCs-NAC (1 mg/mL) and NAC (1 mg/mL).

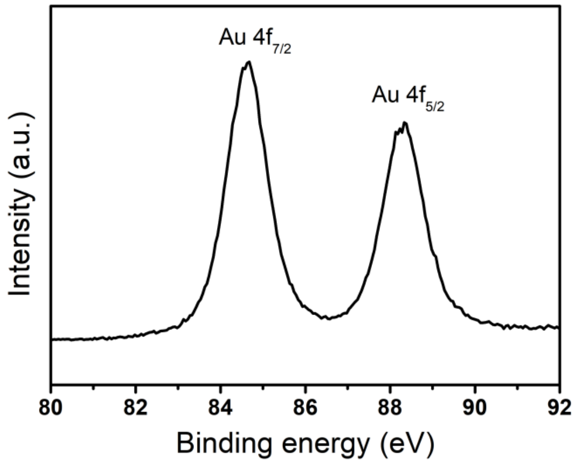

Figure S6. Au 4f XPS spectrum of Au NCs-NAC.

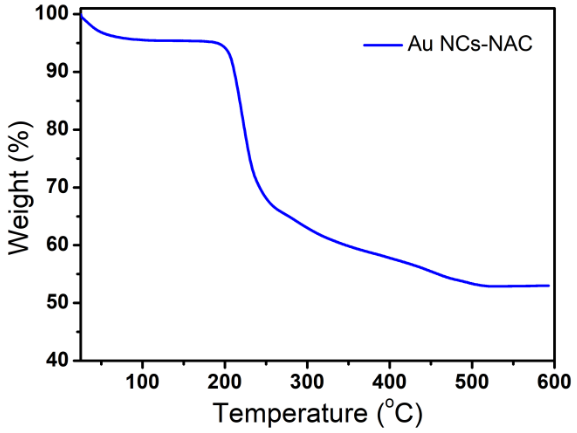

Figure S7. TGA curve of Au NCs-NAC.

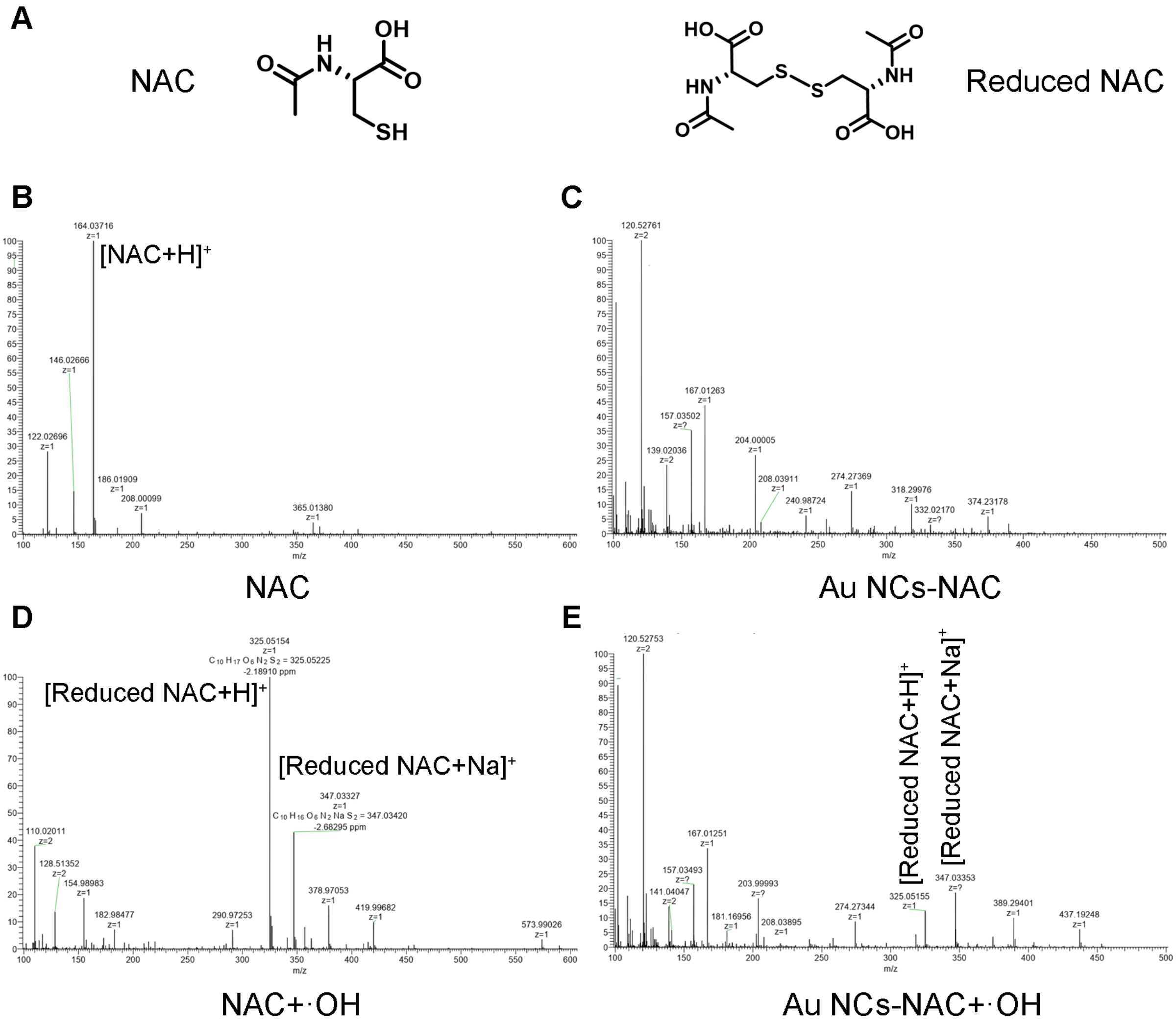

Figure S8. (A) Chemical structure of NAC and reduced NAC. ESI-MS spectrum of (B) NAC, (C) Au NCs-NAC, (D) NAC+·OH, and (E) Au NCs-NAC+·OH.

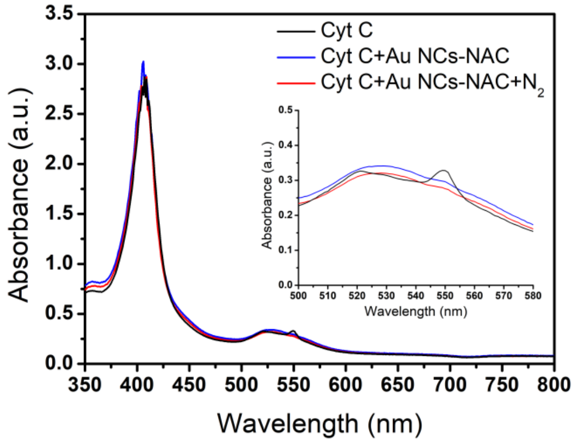

Figure S9. Investigation of electron transfer between Au NCs-NAC and cytochrome C (Cyt c) by Cyt c assay.

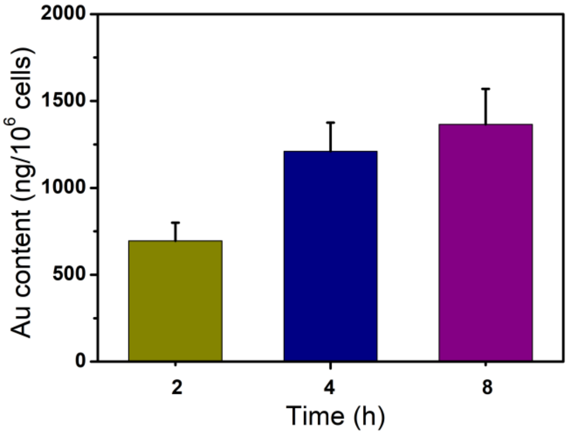

Figure S10. The intracellular uptake of Au NCs-NAC at different time points.

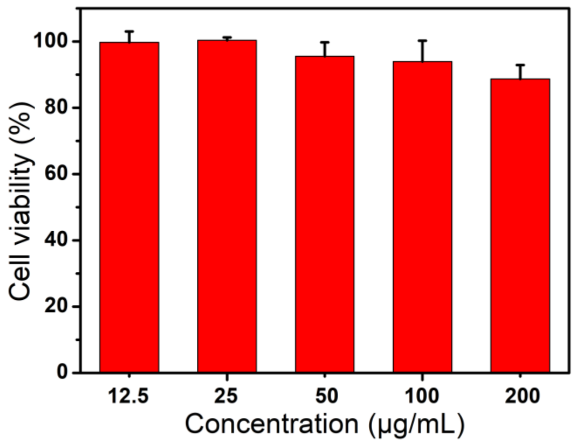

Figure S11. Cell viability of HEK293T cells after treated with various concentrations of Au NCs-NAC.

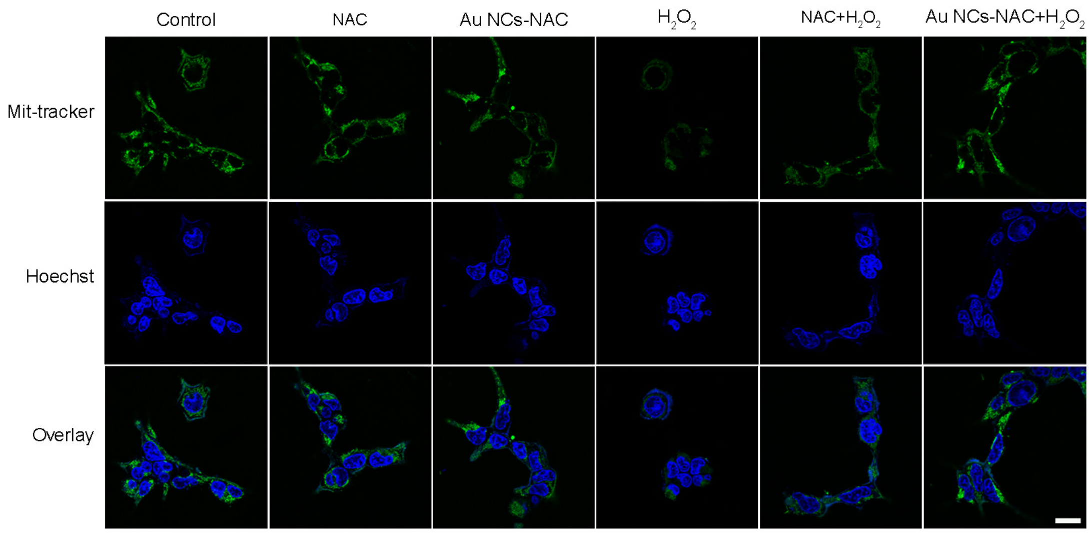

Figure S12. Fluorescence images of HEK293T cells by mitochondrial staining after different treatments. The scale bar is 10 μm.

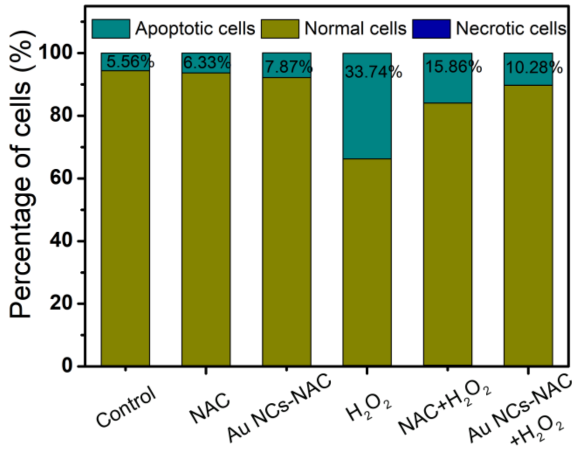

Figure S13. Percentage of cells stained with PI/Annexin V-FITC under different conditions.

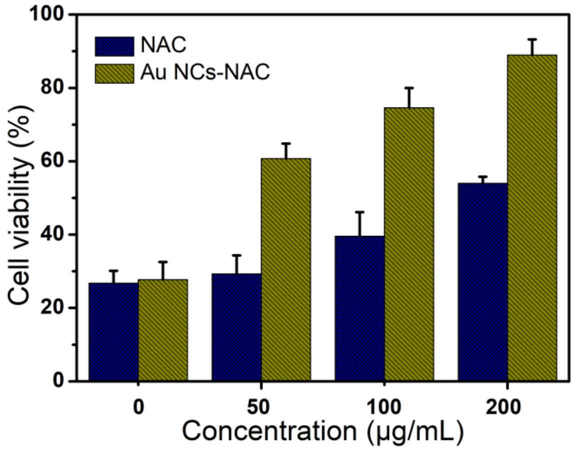

Figure S14. Cell viability of HEK293T cells incubated with LPS when pre-treated with NAC or Au NCs-NAC.

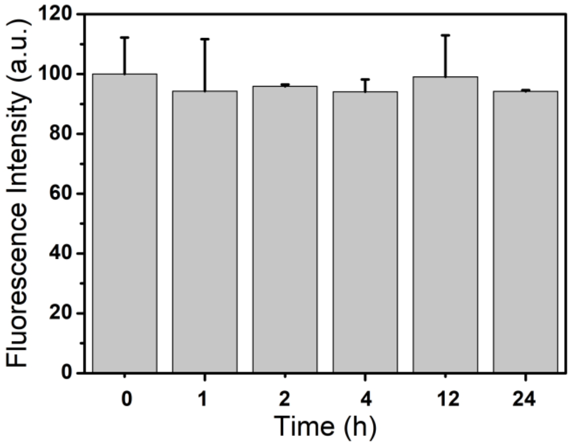

Figure S15. Fluorescence intensity of IR800 labeled Au NCs-NAC.

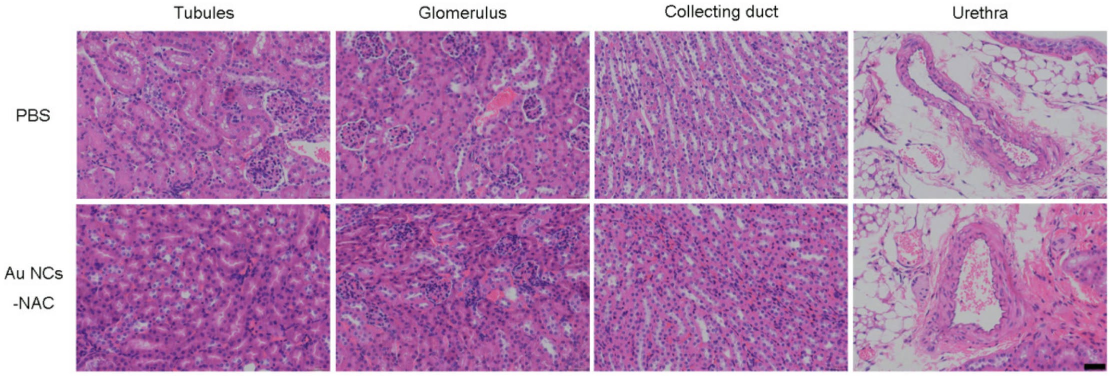

Figure S16. H&E stained images of kidney after treated with PBS or Au NCs-NAC. The scale bar is 100 μm.

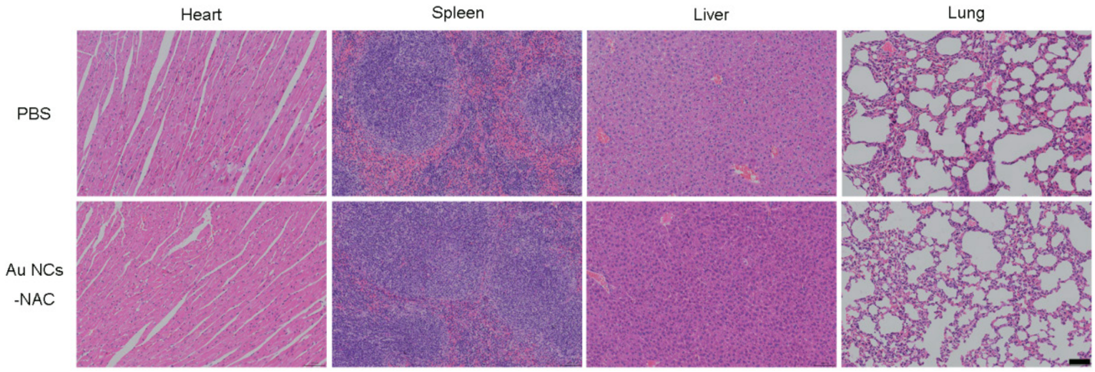

Figure S17. H&E stained images of organs (heart, spleen, liver and lung) after treated with PBS or Au NCs-NAC. The scale bar is 100 μm.

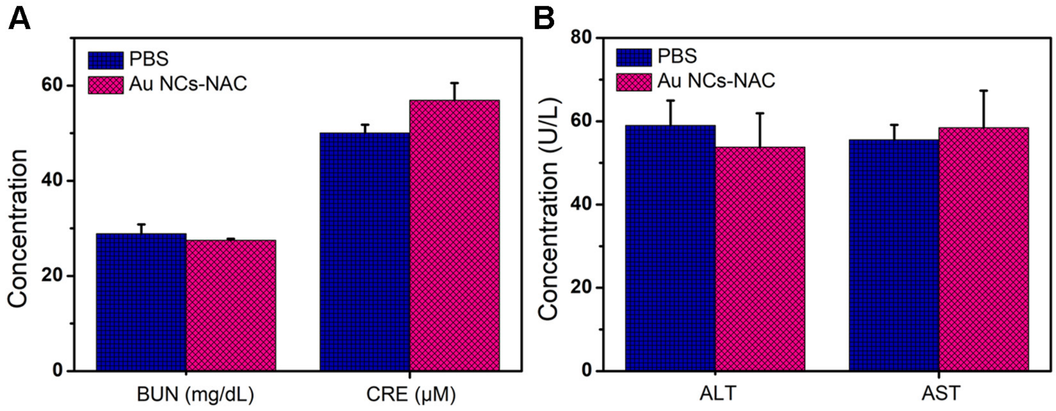

Figure S18. The levels of (A) BUN/CRE and (B) ALT/AST in the serum of normal mice after treated with PBS or Au NCs-NAC.

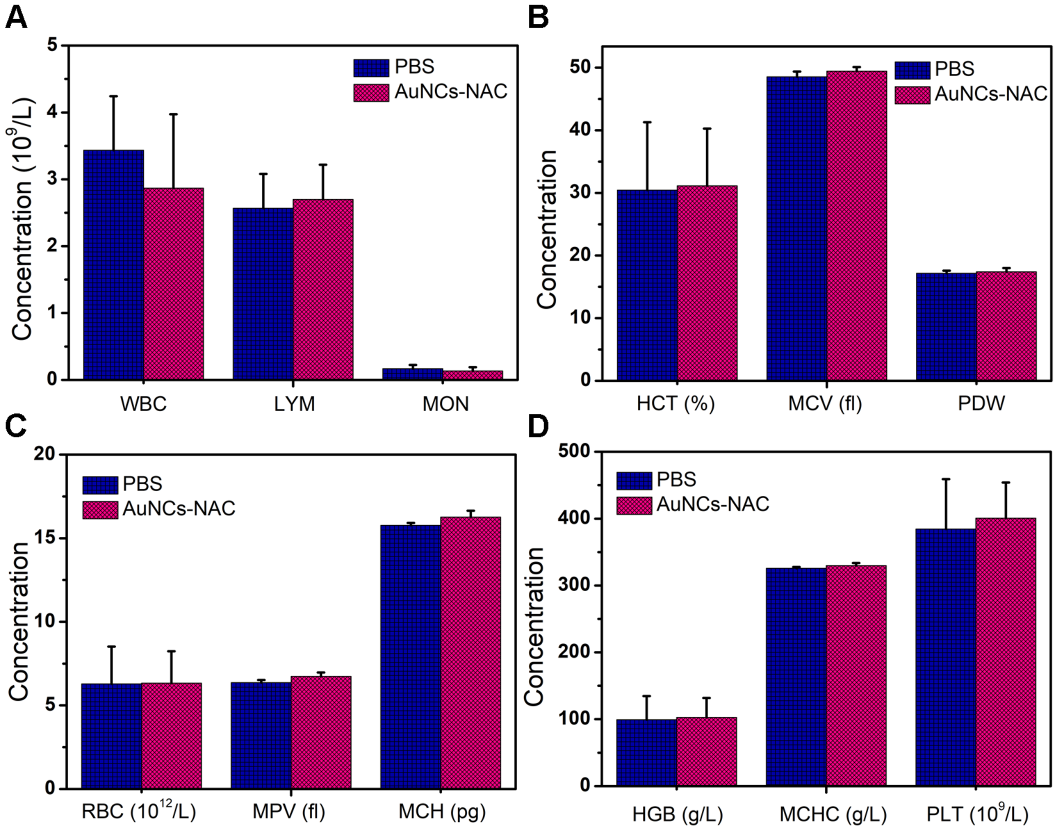

Figure S19. (A-D) Blood biochemical analysis of normal mice after treated with PBS or Au NCs-NAC.

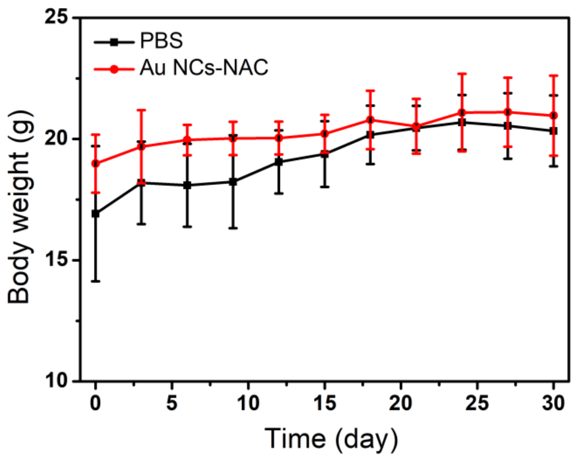

Figure S20. Body weight of normal mice after treated with PBS or Au NCs-NAC.

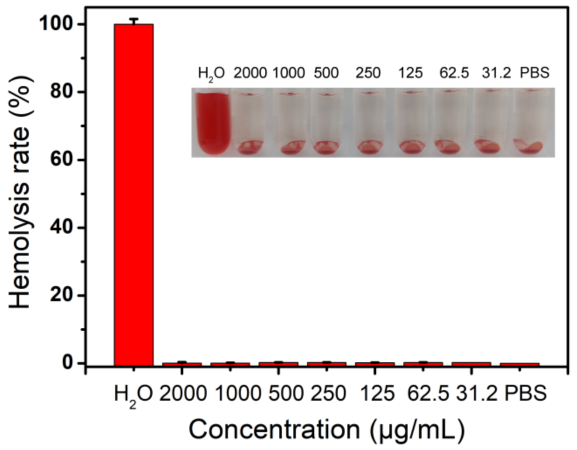

Figure S21. The hemolysis rate of Au NCs-NAC on red blood cells.

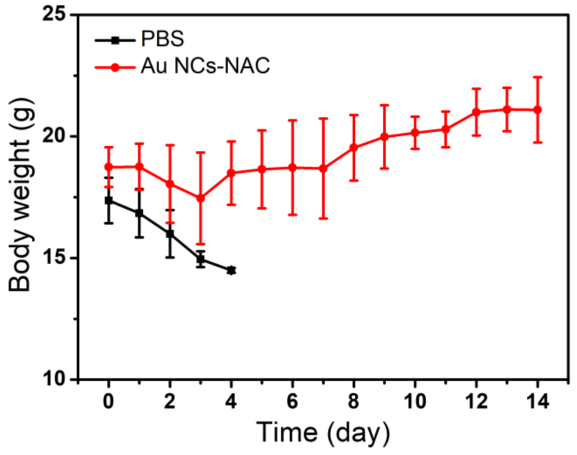

Figure S22. Body weight of AKI mice after treated with PBS or Au NCs-NAC.

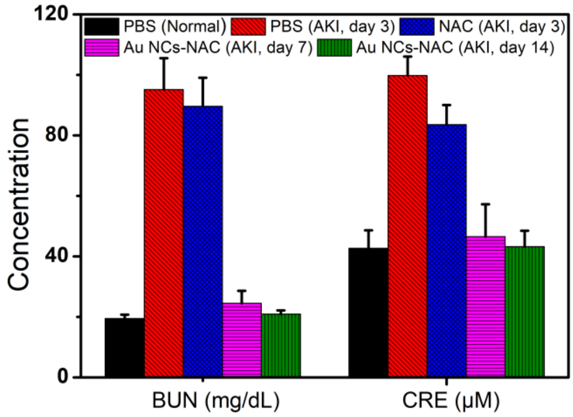

Figure S23. BUN and CRE levels in serum of normal/AKI mice after treated with PBS, free NAC or Au NCs-NAC.

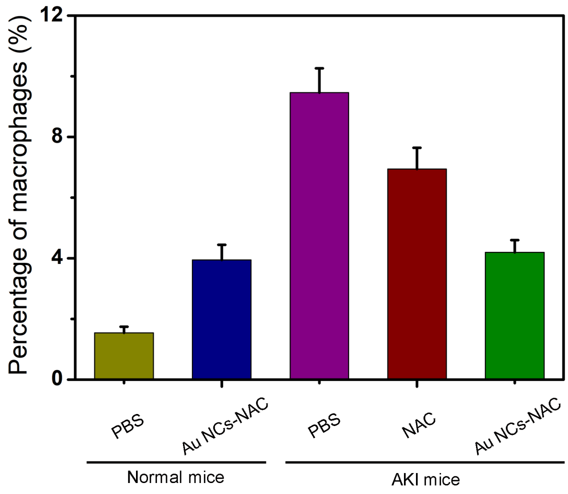

Figure S24. The percentage of macrophages in renal tissues after different treatments.
